# Supplementary material for: Circulating Angiogenic Factors and Ischemic Diabetic Foot Syndrome Advancement—A Pilot Study
Source: Biomedicines. 2023 May 27;11(6):1559. doi: 10.3390/biomedicines11061559 (PMC10295465; doi:10.3390/biomedicines11061559)
Supplement: Supplementary file 1 [file biomedicines-11-01559-s001.zip › biomedicines-2387634-supplementary.pdf]

Table S1. Comparison of clinical characteristics according to ulcer depth. Data are presented as mean (SD) unless otherwise indicated.

Variable	Limited [1] (n = 20)	Penetrating [2] (n = 21)	*p* Value
Age, years	68.75 (10.03)	65.86 (7.51)	0.30
BMI, kg/m^2	27.39 (3.27)	27.70 (3.71)	0.79
Male, %	16 (80.0%)	18 (85.7%)	0.70
Concomitant disorders			
CAD, %	11 (55.0%)	4 (19.0%)	0.02
Hypertension, %	18 (90.0%)	19 (90.5%)	1.00
Prior MI, %	6 (30.0%)	1 (4.8%)	0.04
CHF, %	6 (30.0%)	4 (19.0%)	0.48
Atrial fibrillation, %	6 (30.0%)	2 (9.5%)	0.13
Prior stroke, %	2 (10.0%)	3 (14.3%)	1.00
CKD, %	3 (15.0%)	1 (4.8%)	0.34
CVD, %	2 (10.0%)	2 (9.5%)	1.00
Medications			
Insulin, %	16 (80.0%)	17 (81.0%)	1.00
Sulphonyl urea, %	2 (10.0%)	5 (23.8%)	0.41
Metformin, %	15 (75.0%)	13 (61.9%)	0.51
SGLT-2 inhibitors, %	6 (30.0%)	4 (19.0%)	0.48
GLP-1 agonists, %	2 (10.0%)	0 (0.0%)	0.23
Anti-thrombotic, %			0.58
LMWH	2 (10.0%)	4 (19.0%)	
NOAC	6 (30.0%)	4 (19.0%)	
VKA	1 (5.0%)	0 (0.0%)	
None	11 (55.0%)	13 (61.9%)	
Anti-platelet, %	11 (55.0%)	13 (61.9%)	0.76
Statin, %	13 (65.0%)	11 (52.4%)	0.53
ACEI, %	11 (55.0%)	10 (47.6%)	0.76
AT1-inh, %	4 (20.0%)	5 (23.8%)	1.00
Betablocker, %	12 (60.0%)	12 (57.1%)	1.00
CCB, %	6 (30.0%)	3 (14.3%)	0.28
Blood count and biochemical tests			
Hemoglobin, g/dL	13.79 (2.10)	12.28 (1.66)	0.01
ln(CRP)	4.37 (0.27)	4.45 (0.26)	0.34
ln(HBA1c)	2.05 (0.18)	2.05 (0.20)	0.89
ln(TC)	1.19 (0.26)	1.34 (0.32)	0.13
ln(LDL)	0.54 (0.38)	0.75 (0.44)	0.12
HDL, mmol/L	1.16 (0.32)	1.07 (0.24)	0.30
TG, mmol/L	0.24 (0.36)	0.47 (0.47)	0.09

[1] Confined to the skin and subcutaneous tissue. [2] Reaching the muscle, tendon, or more profound. BMI—body mass index, CAD—coronary artery disease, MI—myocardial infarction, CHF—chronic heart failure, CKD—chronic kidney disease, CVD—chronic venous disease, SGLT-2—sodium-glucose co-transporter-2, GLP-1—glucagon-like peptide 1, LMWH—low molecular weight heparin, NOAC—novel oral anticoagulants, VKA—vitamin K antagonists, ACEI—angiotensin-converting-enzyme inhibitors, AT1-inh—angiotensin II receptor inhibitors, CCB—calcium channel blockers, CRP—C-reactive protein, HbA1c—glycated hemoglobin, TC—total cholesterol, TG—triglyceride, HDL-C—high-density lipoprotein cholesterol, and LDL-C—low-density lipoprotein cholesterol.

Table S2. Comparison of angiogenic factors and clinical characteristics according to patient ages.

Variable	≤68 Years (n = 21)	>68 Years (n = 20)	Total (n = 41)	*p* Value
VEGF-A, pg/mL				0.38

mean (SD)	61.82 (91.38)	40.72 (55.48)	51.53 (75.84)	
median (IQR)	26.20 (8.88, 48.88)	13.60 (2.84, 59.70)	19.07 (3.67, 55.64)	
VEGF-R2, pg/mL				0.36
mean (SD)	12,788.52 (3024.01)	12,005.99 (2376.86)	12,406.80 (2722.62)	
median (IQR)	12,353.93 (10,539.92, 14,688.77)	116,76.19 (10,673.62, 12,893.66)	11,927.98 (10,539.92, 13,801.05)	
PIGF, pg/mL				0.71
mean (SD)	13.16 (2.69)	13.55 (3.92)	13.35 (3.31)	
median (IQR)	12.16 (10.54, 15.51)	12.75 (11.59, 15.54)	12.28 (10.83, 15.51)	
PDGF-BB, pg/mL				0.31
mean (SD),	448.92 (454.95)	334.05 (209.71)	392.89 (357.43)	
median (IQR)	309.49 (240.10, 469.20)	255.16 (165.02, 473.09)	299.64 (215.23, 471.75)	
FGF-2, pg/mL				0.02
mean (SD)	8.83 (5.78)	5.26 (3.43)	7.09 (5.06)	
median (IQR)	7.00 (3.52, 13.97)	5.52 (1.78, 7.75)	6.07 (3.52, 9.42)	
PEDF, ng/mL				0.87
mean (SD)	14,499.11 (7739.90)	14,160.51 (5258.51)	14,333.94 (6566.36)	
median (IQR)	13,527.76 (9720.83, 17,578.05)	14,000.98 (10,647.08, 17,939.33)	13,870.45 (9720.83, 17,802.82)	
ANG-1, pg/mL				0.55
mean (SD)	4284.13 (5995.44)	3404.20 (2552.19)	3854.90 (4611.39)	
median (IQR)	2744.49 (1661.96, 4006.75)	2762.01 (1601.99, 4091.81)	2744.49 (1661.96, 4032.60)	
SINBAD, points				0.13
mean (SD)	4.57 (1.29)	4.00 (1.08)	4.29 (1.21)	
median (IQR)	5.00 (4.00, 5.00)	4.00 (3.00, 5.00)	5.00 (3.00, 5.00)	
WIfI, points				0.68
mean (SD)	5.62 (1.86)	5.40 (1.43)	5.51 (1.65)	
median (IQR)	6.00 (4.00, 7.00)	5.50 (4.00, 6.25)	6.00 (4.00, 7.00)	
ABI				0.25
mean (SD)	0.78 (0.50)	1.04 (0.88)	0.91 (0.71)	
median (IQR)	0.67 (0.58, 0.96)	0.73 (0.45, 1.04)	0.72 (0.46, 1.00)	
TBI				0.76
mean (SD)	0.16 (0.13)	0.18 (0.09)	0.17 (0.12)	
median (IQR)	0.15 (0.06, 0.22)	0.16 (0.13, 0.20)	0.15 (0.11, 0.21)	
tcpO2, mmHg				0.87
mean (SD)	17.00 (12.61)	17.65 (11.95)	17.32 (12.13)	
median (IQR)	12.50 (9.00, 25.25)	15.00 (10.00, 22.75)	14.50 (9.75, 25.25)	
LDF, PU				0.32
mean (SD)	12.36 (4.44)	13.80 (4.73)	13.06 (4.59)	
median (IQR)	11.00 (9.00, 15.00)	13.75 (9.75, 16.25)	12.00 (9.00, 16.00)	
HbA1c, %				0.02
mean (SD)	8.47 (1.72)	7.30 (1.27)	7.90 (1.61)	
median (IQR)	8.10 (7.30, 9.50)	6.80 (6.47, 7.93)	7.50 (6.70, 8.90)	
TC, mmol/L				0.46
mean (SD)	3.58 (1.05)	3.85 (1.27)	3.71 (1.16)	
median (IQR)	3.50 (2.70, 3.90)	3.55 (2.80, 4.60)	3.50 (2.80, 4.50)	
CRP, mg/L				0.74
mean (SD)	19.13 (30.88)	22.56 (31.03)	20.80 (30.57)	

median (IQR)	4.16 (1.79, 11.75)	13.55 (4.71, 22.38)	5.61 (2.95, 15.40)

CRP—C-reactive protein, HbA1c—glycated hemoglobin, TC—total cholesterol, VEGF-A—vascular endothelial growth factor A, VEGF-R2—vascular endothelial growth factor receptor 2, FGF-2—fibroblast growth factor 2, PlGF—placental growth factor, PDGF-BB—platelet-derived growth factor-BB, PEDF—pigment epithelium-derived factor, Ang-1—angiopoietin-1, ABI—ankle–brachial index, TBI—toe–brachial index, tcpO2—transcutaneous oximetry test, and LDF—laser Doppler flowmetry.
